# Physiology

**Published:** 1860-01

**Authors:** S. Weir Mitchell

**Affiliations:** Lecturer on Physiology in the Phila. Med. Association


### PHYSIOLOGY.

BY S. WEIR MITCHELL, M.D.,

LECTURER ON PHYSIOLOGY IN THE PHILA. MED. ASSOCIATION.

### I.—*On the Action of Salts upon the Red Corpuscles of the Blood while in Circulation.* By Dr. Batkin Mascau.

The mesentery of the frog was found most convenient for watching the influence of these agents, partly owing to the want of pigment, and partly by reason of the superficial bifurcation of the blood-vessels. A drop of solution of chloride of sodium (15 per cent.) being added, a change in the circulation is remarked previous to any narrowing of the caliber of the arteries or veins. The interspaces between the single blood-corpuscles disappear, the corpuscles being interrupted in movement and irregularly round. In some of the smallest vessels a complete plugging up occurs, while in neighboring larger ones the circulation is obviously accelerated. In a few moments the smaller vessels plugged up begin to show a movement which extends to them from the vessels still retaining blood-movement, so that they gradually become freed from their plug of corpuscles, and in about half an hour completely resume their usual condition. If, after the formation of such a plug, we cover the preparation with water, the process is immediately arrested; and, on again adding some of the saline solution, the above-described changes in the blood-vessels extend to all the capillaries in the field of observation, and the capillaries of larger diameter become also stopped up. The changes are finally observable in the veins and arteries, and in the last also an evident pulsation is seen.

After some hours, on being left to itself, the circulation becomes re-established, (firstly in the large, and then in the small vessels,) but not in its original rapidity.

A stasis so produced can easily be dissipated by the addition of water; but if some of the saline solution be added in its place, the circulation in the arteries immediately ceases, probably owing to interruption of the communication between the arteries and veins by means of arrest in the capillaries. At the same time a starting movement begins in the veins, by which at each systole the blood-corpuscles move from the periphery toward the centre, and at the commencement of the diastole recede in the opposite direction. Finally, this starting movement in the veins passes into an unbroken stream from the centre to the periphery, at first being very rapid, and then, becoming slower and slower, altogether ceases. The veins and capillaries then appear to be filled with blood, while in the arteries the movement continues.—(*Virchow's Archives*, Band xv., Hefte 1 and 2, p. 173.—*British and Foreign Med.-Chir. Review*, October, 1859.)

II.—*Normal Presence of Alcohol in the Blood.* By WM. HUTSON FORD, M.D.

The author of this elaborate and ingenious paper prefaces his experiments by a long argument, in which he starts with the premise that liver-sugar constantly formed is poured into the blood and disappears in the lungs. He combats at some length the idea that the sugar thus lost to chemical view is converted into lactic acid, and is of opinion that he can show that it is really changed into alcohol. The most valuable and interesting part of the essay is, of course, the experimental proof of the above proposition offered by the author.

He proposes to prove that the sugar of the liver is converted into alcohol, and in this form ministers to the calorific functions of the animal economy.

It will naturally occur to most of our readers, that of late the researches of Pavy have rendered it somewhat doubtful whether sugar is ever formed by the healthy liver. However this may be, if Dr. Ford's researches have been conducted with such care as to insure the accuracy of his experiments, their proof of the existence of alcohol in the blood will be of such value that the failure of the author's inductive process, owing to defective premises, will be of but little moment. The author who proves the existence in the blood of an unsuspected element, such as alcohol, may well be excused a defect in reasoning, especially when this is due not to carelessness, but to a too great faith in the conclusions of others. As a large part of the value of any novel labor of this kind must depend upon the number and character of the precautions with which the author surrounds his experiments, we shall detail the means employed by Dr. Ford in the present case, and with such detail as may afford grounds for judging of the probable character and accuracy of his experimental results.

"The only known method of separating alcohol from aqueous fluids in which it is contained is *distillation.* In conjunction with this process, we have employed eliminative agents, by which a weak, spirituous fluid, sometimes containing little else than water and alcohol, is obtained with certainty, when this latter body was originally present. Distillation is, moreover, the proper method for experimentation of this kind, on account of the completeness and promptness with which alcohol is separated, and the alcoholic fermentation arrested by coagulation of albuminous principles consequent upon the use of heat. We have distilled blood, lung tissue, liver tissue, and pancreatic tissue, in various conditions; always without previous admixture of substances designed to separate albumen, dissolve blood-corpuscles, or destroy sugar. Tedious processes of filtration, unavoidable in such methods, would occupy much time, and lessen the significance of the experiment by allowing a possible formation of alcohol from the hepatic sugar present. Coagulation of albumen by heat would, of course, dissipate any alcohol the liquid might contain. We have, for these reasons, except in certain cases, invariably submitted organic matters to the distillatory process, without prior admixture, upon the bath of chlorides of sodium or calcium, of sand, or upon the furnace itself; and when chemical substances have been mingled with blood before distillation, they have not been intended to effect chemical changes in that fluid, nor has their employment in any respect lengthened the period of time elapsing between *the death of the animals and the elevation of their structures to* 212° F. The apparatus used has varied somewhat with the nature of the experiments. For organic matters, copper balloons, holding from two to sixteen quarts, have been employed." For obvious reasons, glass retorts could not be used, except where corrosive reagents were to be added to the boiling mass. It will give some conception of Dr. Ford's industry and of the difficulties of his task, when we state that fifteen or more successive distillations were usually required to concentrate the distillates to the proper extent. Every possible precaution usual in such research seems to have been employed by Dr. Ford, and so far as a careful examination of his statements authorizes the formation of an opinion, no fault can be found with the details of the manipulations resorted to.

"The organic substance to be examined is weighed and introduced into a copper balloon of peculiar shape. The amount used should never more than two-thirds fill this vessel, and when blood is acted on it ought to occupy but one-sixth of the receiver. A strong heat is next applied by the aid of a salt or sand bath, or, in some cases, a furnace fire."

"When the bath is used, the first distillate is transparent; but when direct heat has been applied, it is whitish, semi-opake, and contains free ammonia, carbonate of ammonia, sulphuretted hydrogen, (especially when decomposing matters are experimented upon,) two or three oily substances, and one concrete oily body, a very volatile fluid coming over before general ebullition, and perhaps some acid substances in union with ammonia; besides a minute quantity of alcohol when this substance has been present in the distilland. The reaction is always alkaline. To this, first, hydrochloric acid is added, and after twelve hours it is redistilled from a glass vessel. Second, the distillate is treated with an excess of a cold saturated solution of acetate of lead; sulphide and chloride of lead fall, and the mixture is filtered to separate them. After a new addition of hydrochloric acid, another filtration, etc., the fluid is boiled, while, from time to time, small quantities of the acid are being added to the distilland.

"The distillate is thus concentrated, and a concrete, shining-yellowish, fatty-looking substance is now seen in the distillate, and is completely removed by the addition of a crystal of nitrate of silver, filtering and washing, as usual. Upon ebullition the distilland becomes dark hued, oxyde of silver and metallic silver falling down. Neither the concrete volatile oil nor the volatile substance, coming over into the tubes just before ebullition, and which we suspect to be acetal or aldehyde, is again observed."

Next follow charcoal purifications and repeated distillations. "The final distillate," says our author, "should weigh from fifteen to thirty grains, should have come over perfectly transparent, must be neutral to test-paper," etc. When the distillations are made with the bath heat alone, no empyreumatic odor is generated during the first process. In the final distillate, Dr. Ford thinks that he found alcohol. His tests of its presence were—

1. Chromic acid dissolved in sulphuric acid, and employed as usual. The emerald green tint, which results when this is added to a mixture containing alcohol, was relied upon as the test. The defects of this test are admitted by the author, who conceives, however, that his processes were such as to exclude all chances of fallacy.

2. Dr. Ford observed that the optical appearances within the glass tubes, when the distilland first began to boil, were so peculiar when alcohol was present as to enable him to recognize it. This test can scarcely be regarded as of much value, although, for the author, long observation may have made it very reliable.

3. When not less than two per cent. of alcohol is present, it can be made to take fire and burn, by proper management, such as Dr. Ford describes.

"This test," he adds, "is entirely conclusive. The primary distillate, and all successive ones, until the very last portion is reached, where we assume alcohol to be present, constantly refuse to burn in the tube, or to color the chromic acid solution. Inflammable empyreumatic substances should, however, have been far more abundant early in the series than in the last distillate. Some organic acids, as formic and acetic, are inflammable in the state of vapor, but only when pure, or nearly so; the greater part, however, of the ultimate distillate was water; and acids are not present, the fluid being neutral to test-paper."

On p. 20, Dr. Ford shows that when alcohol has been artificially mingled with organic matters, it may be recovered by the above processes, and is recognizable by the same tests. He also shows that the physical characters of the last distillate indicate the presence of alcohol. No ultimate analysis of the supposed alcohol has yet been attempted.

Dr. Ford's direct experiments were as follows: After quoting Bernard, to show that sugar disappears from blood exposed to various gases, and even to the air, he describes five experiments, in all of which he examined ox blood for sugar, and, after finding it in each case, he exposed the blood to the air or to carbonic acid gas for from two to four days. On distillation he obtained alcohol from every specimen, and hence affirms that in these specimens the *normal* sugar of the blood had passed into alcohol.

In experiments six and seven, the presence of alcohol in putrefying liver was determined.

In experiments eight to eleven, inclusive, lung tissue was hashed, set aside to putrefy or alter, and finally examined for alcohol by distillation. It was found in every case. From these results, Dr. Ford infers the pre-existence of sugar in the lung tissue.

In all the preceding instances, if sugar were present, the alcohol was to be regarded as derived from it, the albuminoid matters present acting, according to our author, as the ferment. But if this process went on in life, we should be able to detect in the fresh tissues not only sugar, but alcohol.

To test this point, fresh lung tissue was distilled, so that but from forty-five to ninety minutes elapsed between the slaying of the animal and the raising of his tissues to a temperature of 212° F. In every case alcohol was detected.

In a third series of experiments, eight in number, blood from the neck-vessels and lungs and heart of the ox were similarly treated, and alcohol so invariably detected as to justify us, Dr. Ford supposes, in regarding it as a normal constituent of the blood. The process employed to collect the blood was such as to give a mixture of various bloods, and although this fluid came chiefly from the lungs, any mixture with other blood can only do harm, and might have been well avoided in experiments otherwise so elaborate.

In experiments twenty-eight, twenty-nine and thirty, it is attempted to be shown that when a known amount of alcohol is added to blood, it disappears in part by "hæmal oxydation."

Finally, alcohol was obtained by distilling fresh pancreatic tissue.

Dr. Ford's conclusions are as follows :—

1. "In blood which is allowed to enter into the putrescent state, sugar, normally present, is destroyed; being changed by the decomposition of albuminous matter into alcohol and carbonic acid, and this whether the blood be extravasated or still within the capillaries of the liver or lungs.

2. "Alcohol exists in liver tissue within *seven* (?) minutes after death.

3. "Sugar is normally and constantly present in lung tissue.

4. "Alcohol exists in lung tissue within fifty-five minutes after death.

5. "Alcohol exists in the mixed thoracic blood of the slaughter-house within forty-five minutes after death.

6. "Alcohol exists during the digestive state, in the pancreatic tissue, sixty minutes after death, and in the jugular blood of a dog in ten minutes after opening the vein.

7. "Alcohol, when added, is destroyed in aerated blood with greater rapidity than in blood saturated with carbonic acid gas."

Dr. Ford's theoretical conclusions are as follows, and although we may entertain more than considerable doubts as to some of them, in justice to the author, and his most industrious and ingenious essay, we give them somewhat in full.

1. The hepatic sugar secreted by the liver begins to ferment in that organ, continuing to do so as it passes through the hepatic veins, vena cava, heart, and lungs; in the fasting state, finishing its fermentation completely before the blood enters the pulmonary veins.

2. That the above fermentation is into alcohol and carbonic acid.

3. That venous blood possesses a greater fermentative power upon sugar than arterial; and that sugar and alcohol are, without exception whatsoever, present *at the same time* for every viscus or part of the body in the blood of such organ or portion, except when all the sugar of a certain blood has been fermented, while the alcohol has not yet been entirely destroyed; it may then be possible to obtain alcohol from a substance in which sugar does not exist; but, in general, in the dissemination of sugar during digestion and at other times, as in exercise, agitation, or experiment, sugar is *universally accompanied by alcohol in the blood.*

4. Alcohol produced by the hæmal fermentation of hepatic sugar is consumed or oxydized by a hæmal combustion, whose ultimate products are carbonic acid and water, and which constitutes a main source of animal heat.

5. That the red corpuscles are the agents of the hæmal oxydation of alcohol, etc.

6. That the ferment is albuminous matter in a state of change, etc.—(*Journal of the Elliott of Natural History*, vol. i., article II., Charleston, S. C., May 15, 1858.)

III.—*Experimental Researches on the Exciting Influence of Light, Cold, and Heat on the Iris in the Five Classes of Vertebrate Animals.* By E. Brown-Séquard, M.D.

From an interesting investigation of this subject, this distinguished observer has arrived at the following conclusions:—

1. Light is capable of acting as an energetic excitant of the iris and of thus producing a narrowing of the pupil.

2. The iris of the fish is not immobile, as was at one time supposed, and the changes in the size of the pupil are in this class more marked in certain cases than in mammals.

3. When light acts on the iris directly, and thus causes contraction of the pupil, it acts principally by virtue of its illuminating rays, and especially by its yellow rays.

4. When light causes contraction of the pupil in eyes removed from the head, it is rather by an action upon the muscles of the iris than upon its nerves.

5. A sufficient change of temperature causes the pupil to contract if dilated, and to dilate if previously contracted, in the eyes of mammals or birds, and after the organ has been removed from the orbit.

6. When an isolated eye is acted on by light, heat, cold, or galvanism, its pupil moves so considerably as to make it impossible to admit that this is due to vascular turgescence.

7. It seems likely, that if light is capable of acting as an excitant of the retina and iris, and not of other nerves or muscles, it is owing to the peculiar arrangement of the ocular tissues in thin layers, which may be an anatomical condition essential to the result.

8. The irritability of the iris remains after that of all the other muscular structures of the economy has departed.—(*Journal de la Physiologie de l'Homme et des Animaux.* Paris, No. vii., 1859.)

IV.—*On the Substance called Animal Starch.* By C. Schmidt.

Since the researches of Virchow on the amylaceous corpuscles so called, they have been studied with the microscope and with chemical reagents used on the microscope field; but to establish their nature as true starchy bodies, it is necessary that they should be shown, on ultimate analysis, to be hydrocarbons, or at least like other amylaceous matters, they should change into glucose when treated with acids in the way usually employed.

The small size of these elements and their union with neighboring tissues is such an obstacle to their analysis that C. Schmidt has been obliged to resort to the following means:—

It is possible, by choosing a tissue containing a large amount of the supposed starch cells, and washing this repeatedly with water, alcohol, and ether, to eliminate the soluble albuminates, fats, leucine, tyrosine, insosite, etc. What remains of an ordinary tissue so washed should yield, on analysis, a certain amount of hydrogen and carbon in union with nitrogen and oxygen, in such proportions as is usual in albuminoid matters, as albumen or fibrin. If, however, an amylaceous matter be present in any considerable amount, it would raise relatively the preparation of carbon, and lower that of the nitrogen. If, at the same time, treatment by sulphuric acid produced in the mass glucose, we should have proof enough that a hydrocarbon had been present in considerable amount, and that it was of a gummy or of an amylaceous character.

Accordingly, tissues rich in the supposed amyloid body were washed with alcohol, ether, and water, and variously treated with sulphuric acid. If there was present any starch, it should have been converted into sugar, a change which, however, did not occur.

Ultimate analysis of the suspected tissues also proved that nitrogen was present in such large amount as to make it evident that the pretended animal amyloid was not a hydrocarbon, free from nitrogen and analogous to cellulose.—(*Jour. de la Phys.*, No. vii., 1859, p. 516.)

V.—*The Capillaries of the Brain.* By CHARLES ROBIN, M.D., etc.

In an extremely interesting memoir upon the peculiarities of structure to be observed in the capillaries of the brain, M. Robin describes minutely a special tunic which surrounds the encephalo-rachidian capillaries.

"A certain number of these little vessels," says the author, "are enveloped by a special coat of the thickness of one or two-thousandths of a millimeter. This layer is homogeneous or slightly striated, is somewhat wavy, and lies at one to three-hundredths of a millimeter from the outer wall of the vessel. The interspace thus left is sometimes full of a colorless fluid mixed with molecular granules and sometimes with small, free nuclei, which are either closely packed or scattered at rare intervals, so as to leave the peculiar oval nuclei of the walls of the capillary in distinct view.

In all cases of persons aged from forty to forty-five years, this interspace also contains fat granules, isolated or in groups, and a large quantity of amorphous granules of hæmatosine, of various sizes, up to the two-hundredths of a millimeter. In no case are these bodies accompanied by blood-globules."—(*Journal de la Physiologie*, etc., No. 8, 1859, p. 543.)

VI.—*Some Observations on the Coagulation of the Blood.* By JOHN DAVY, M.D.

From a number of experiments on the blood of the common fowl, obtained by dividing the great vessels of the neck, and selected because the blood of birds exhibits the phenomenon in question in the most striking and rapid manner, the author infers:—

1st. That there are no indications afforded of the escape of volatile ammonia during the coagulation of the blood of the fowl, or of the presence of this principle in the blood.

2d. That the addition of ammonia in a notable quantity does not prevent coagulation.

3d. That a sudden reduction of the temperature of the blood, even when fully exposed to the air, has a greater influence in retarding the coagulation than the assigned cause of its fluidity, volatile alkali, when added.

The few trials made by the author with the sesquicarbonate and neutral carbonate of ammonia tend to show that "these salts of ammonia, used in small quantities, have the effect of retarding coagulation. In this their influence is analogous to that of most salts of the alkalies, and, like them, we know that in a large proportion they prevent it, and yet that, on dilution with water, coagulation takes place."

Some further experiments, also made with the view of testing the hypothesis advanced, that the presence of ammonia in the blood is the cause of its fluidity, are detailed. These are designed to show that "even on the supposition of ammonia existing in the blood in a volatile form, no appreciable quantity can escape in the short period of two or three minutes, which is about the time required for the coagulation of the blood of the fowl." And that "the volatile alkali in its action on fibrin is chiefly remarkable for rendering it viscid, and that its solvent power is inconsiderable."

Dr. Davy, in conclusion, observes that, as his results "are opposed to the influence of the coagulation of the blood being owing to the escape of any volatile matter, and *a fortiori* of the volatile alkali, the existence of which in the blood remains to be proved, they leave the phenomenon, as hitherto, a problem for solution, and open to question whether it be the result of loss of vitality or a chemical result depending on a new arrangement of elements of the coagulating part, the fibrin, without, as regards their number, any change of their sum."

It will be seen from the above that Dr. Davy does not accept Dr. Richardson's views as to the cause of the coagulation of the blood.—(*Dublin Quarterly Journal of Medical Science*, November, 1859, p. 425.)

